# Geographic variation in Alzheimer’s disease mortality

**DOI:** 10.1371/journal.pone.0254174

**Published:** 2021-07-01

**Authors:** Michael Topping, Jinho Kim, Jason Fletcher

**Affiliations:** 1 Departments of Sociology, University of Wisconsin-Madison, Madison, Wisconsin, United States of America; 2 Center for Demography of Health and Aging, University of Wisconsin-Madison, Madison, Wisconsin, United States of America; 3 Department of Health Policy and Management, Korea University, Seoul, Republic of Korea; 4 Interdisciplinary Program in Precision Public Health, Korea University, Seoul, Republic of Korea; 5 La Follette School of Public Affairs, University of Wisconsin-Madison, Madison, Wisconsin, United States of America; 6 Agricultural and Applied Economics, University of Wisconsin-Madison, Madison, Wisconsin, United States of America; 7 Population Health Sciences, University of Wisconsin-Madison, Madison, Wisconsin, United States of America; German Centre for Neurodegenerative Diseases Site Munich: Deutsches Zentrum fur Neurodegenerative Erkrankungen Standort Munchen, GERMANY

## Abstract

**Objective:**

Accumulating evidence suggests the possibility that early life exposures may contribute to risk of Alzheimer’s Disease (AD). This paper explores geographic disparities in AD mortality based on both state of residence in older age as well as state of birth measures in order to assess the relative importance of these factors.

**Methods:**

We use a subset of a large survey, the NIH-AARP Diet and Health Study, of over 150,000 individuals aged 65–70 with 15 years of mortality follow-up, allowing us to study over 1050 cases of AD mortality. We use multi-level logistic regression, where individuals are nested within states of residence and/or states of birth, to assess the contributions of place to AD mortality variation.

**Results:**

We show that state of birth explains a modest amount of variation in AD mortality, approximately 4%, which is consistent with life course theories that suggest that early life conditions can produce old age health disparities. However, we also show that nearly all of the variation from state of birth is explained by state of residence in old age.

**Conclusions:**

These results suggest that later life factors are potentially more consequential targets for intervention in reducing AD mortality and provide some evidence against the importance of macro-level environmental exposures at birth as a core determinant of later AD.

## Introduction

Place-based differences in later life mortality and health are a well-documented and increasing body of literature [1,2]. While life expectancy has improved for decades, there still remains a gap of 7.5 years across states [3]. Existing research has predominantly focused on individual level factors in health outcomes but have increasingly included information on the influence of place [4]. Research has found that an individual’s place of birth is connected with the development of chronic illnesses later in life such as cancer, diabetes, and heart disease [5–7]. Contextual factors also are considered as principal indicators of population health, but when and how these contexts come into play is not fully understood. Additionally, little is known on how early environmental factors affect mortality later in life.

The Developmental Origins of Adult Heath and Disease (DOHaD) and life course frameworks state that health disparities in later life could be a result of place-based exposures throughout the lifespan, not just current exposures [8]. The impact that these life exposures have can both accumulate into later life and influence other health outcomes [9–11]. This accumulation of risk across the life course, rather than at a particular stage, may be what is ultimately detrimental for individual’s health and well-being [12].

The past and present exposures individuals are subject to can be distinguished by migration. About one third of individuals in the United States live in a state in which they were not born in [13]. Therefore, a significant portion of the inequalities in health and mortality might be linked to exposures that people encountered early in life, opposed to a later period in the life course. Nevertheless, since mortality is often examined through the lens of the late period of one’s life, little consideration is given to the exposures early in an individual’s lifespan.

Past research has consistently documented key social conditions of health that persist throughout the life course and impact individual mortality risk [14]. Looking at the specific effects that state residence contexts have on mortality and health in adulthood, they typically examine a myriad of predictors, such as socioeconomic status, policy, social capital, and income inequality [15,16]. Conversely, other studies have examined the effects of state of birth on health outcomes in early life. State-level policies on poverty reduction and health care coverage have been shown to have an impact on infant health, such as birth weight [17,18]. Such poor health outcomes in early life have the potential to leave long lasting effects on lifetime health [19,20]. Moreover, unhealthy environments in early life typically can lead to challenges in learning, being employed, or have earning capability [21,22]. In turn, these difficulties undoubtedly have significant ramifications for health, well-being, and mortality in later life.

One poor health outcome that individuals encounter later in life is Alzheimer’s disease (AD). AD is a debilitating disease which shortens life expectancy, impairs memory, and is a key cause of physical disability and lower quality of life to older adults [23–25]. Since occurrence of AD is associated with an increase in age [26], it is presumed that the disease will have a higher prevalence due to greater population aging in society. Thus, there is a fundamental need to attempt to ascertain the determinants of the disease that is the sixth leading cause of death in the United States [27].

However, despite the prevalence of AD, few studies have attempted to connect early life exposures with mortality from the disease later in life. This is because a great deal of mortality studies in the United States often lack the ability to consider exposures at different points throughout the life course. Moreover, AD mortality studies often examine the period from the diagnosis to the point of death because it is a progressive disorder [24]. Related research focuses on either demographic or clinical factors that show a higher likelihood of mortality of those who already have the disease [28,29].

AD prevalence and mortality have been associated with a growing set of factors: race/ethnicity [25,30], educational attainment [31], nutrition [32], mental health [33], and air pollution [34,35]. While all of the factors outlined are indeed significant determinants of health, there is little literature on early life contexts and their impact on AD mortality. Moreover, there is no existing research regarding the association of one’s state of birth with AD mortality. As a crude measure of early life exposure, state of birth could provide an omnibus test of whether variation in early environments across the US are consequential for developing AD later in life.

This study is unique in the sense that it takes advantage of data that can link information regarding state of birth and state of residence for a large and representative sample of individuals in the United States. Most other research is limited to smaller convenience samples that are not well powered to examine state-level variation in AD mortality. Specifically, this paper addresses the following: (1) the impact that early environments and contexts contribute to differences in AD mortality and (2) what impact state of birth effects differ by social categories such as race/ethnicity, sex, and educational attainment. Ultimately, the aim of this paper is to increase the understanding of the critical determinants of health and health behaviors and effect that early environments have regarding outcomes throughout the life course.

## Materials and methods

### Data

This data utilized in this research was taken from the NIH-AARP Diet and Health Study (DHS). The DHS is a large prospective cohort from members of the American Association of Retired Persons (AARP), ranging from 50 to 71 years old, who responded to a mailed questionnaire between 1995 and 1996 [36]. Initially, over 3.5 million members of the AARP were mailed the questionnaire, resulting in 620,000 responses. Of those responses, nearly 570,000 provided data that was usable for analysis. The participants of this study were from six states (California, Florida, Louisiana, New Jersey, North Carolina, and Pennsylvania) and from two cities (Atlanta, Georgia and Detroit, Michigan) in the US. All cohort participants signed a written informed consent at enrollment, and the study protocol was approved by Special Studies Institutional Review Board of the National Cancer Institute.

DHS asked a comprehensive questionnaire which assessed lifestyle factors and diet of the participants at baseline. Specifically, the questionnaire addressed information on nutrient intake, along with health questions, family illness history, and other health-related conditions. Demographic information was collected from participants as well, such race/ethnicity, sex, and educational attainment, along with other variables to measure health outcomes and well-being. This source of data is unique due to its large sample sizes, which is essential in order to examine the rare outcome of AD mortality. Moreover, it is necessary to have large sample sizes in order to appropriately examine state-level variation. Other datasets (e.g., Health and Retirement Study) that include the variables laid out in this study are rather limited, due to the much smaller number of cases of AD mortality.

**Fig 1:**
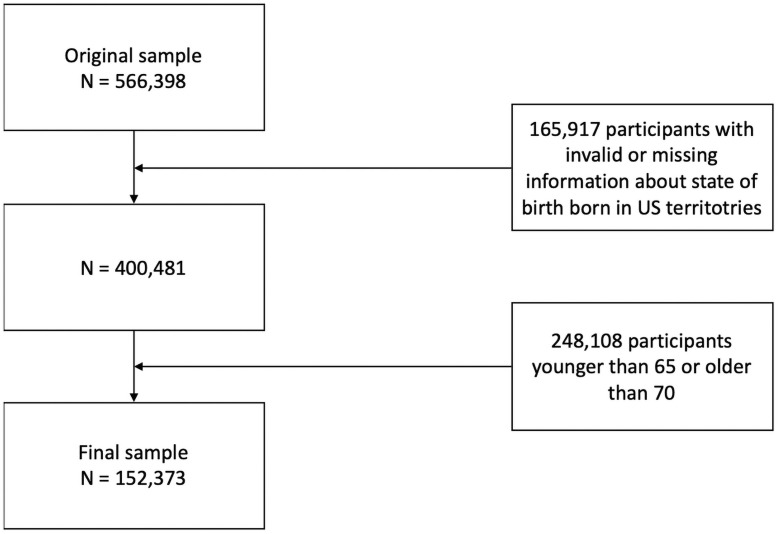
A flow chart of the study sample.

Starting from the initial 566,398 respondents in the original study, specific observations were dropped. First, of the original sample, we dropped 165,917 with invalid states of birth, or those born in United States territories (American Samoa, Guam, Puerto Rico, and the Virgin Islands), and those with missing values. Specifically, the observations dropped with missing values were about missing social security numbers, which would have prevented state identification in our analysis. Next, in order to focus on individuals at higher risk of experiencing AD mortality over a 15-year follow-up, a further 248,108 observations were dropped so that the sample is of individuals between the ages of 65 and 70. The final sample that was used for statistical analysis was 152,373 (Fig 1). We found that older adults are more likely to be in the analytic sample and those of other race groups are less likely to be in the analytic sample than non-Hispanic whites (S1 Table). Despite this, our final sample size provides more statistical power than alternative datasets available like the Health and Retirement Study, with about five times the amount of observations to examine [37].

### Measures

#### AD mortality

The key outcome variable in this study is AD mortality, which is ascertained from the follow-up of the DHS. The vital status of the individual was obtained by the annual linkage of the cohort in the DHS to the Social Security Administration Death Master Files in the US verification of vital status [38]. Cause of death information will then come from follow-up searches of the National Death Index, focused on death from AD (ICD-10 Code G30). Our data is linked to the NDI through 2011, allowing 15 years of follow up.

#### State of birth

State of birth was created by using the first three digits of the individual social security number of each in the cohort (asked in initial survey), allowing information by state and year to be obtained [39].

#### State of residence

State of residence was ascertained from the original survey, which asked the participants which state they lived in at the time. The eight states of residence reflected in this study are: California, Florida, Georgia, Louisiana, Michigan, New Jersey, North Carolina, and Pennsylvania.

#### Covariates

Sex was used as a variable to account for the difference in mortality risk that exists between men and women. Likewise, race and ethnicity (non-Hispanic white, black and others) were utilized to account for the differential risk that exists along the boundaries of race and ethnicity. Educational attainment levels (less than high school, high school, some college, and college grade or more) were included to account for the influence that schooling provides throughout the life course with regard to health outcomes. Age is included as a covariate as well, specifically those that are between the ages of 65 and 70 years old to see the differences in health outcomes are impacted later in the life course of individuals. It is worth noting that we did not control for personal characteristics, such as physical and mental health status and functional status, because those characteristics are likely mechanisms, not confounders of state of birth.

### Analytic strategy

In this study, a series of multi-level logistic regression models are used with state of residence and/or state of birth random effects. The full model is specified as the following equation:

$$\text{ln}\;\left(\frac{p_{ijk}}{1-p_{ijk}}\right)=b_{0}+b_{1}{age}_{i}+b_{2}{sex}_{i}+b_{3}{race/ethnicity}_{i}+\varepsilon_{j}+\varepsilon_{k}+\varepsilon_{ijk}$$

where $\varepsilon_{j}\sim N\left(0,\sigma_{j}^{2}\right)$ and $\varepsilon_{k}\sim N\left(0,\sigma_{k}^{2}\right)$. In the model, *b*_0_ is the average log-odds of mortality among individuals in the sample, *ε*_*k*_ is the effect of state of birth and *ε*_*j*_ is the effect of state of residence. Both are assumed to be normally distributed. *ε*_*ijk*_ is the error term at the individual level. We control for a limited set of factors in order to not “over control” for the effects of state of birth on later life AD mortality. Our age control removes composition differences between the states.

The first and second model in the series includes state of birth random effects, with the second of the two incorporating all fixed effects. Then, the third and fourth run identical analyses, but with state of residence random effects, with the fourth including all fixed effects. The fifth and final model analyzes all fixed and random effects, in order to see differences between state of residence and state of birth variations.

The main results are first presented and include analysis from all states. Supplementary tables provide subsequent results that display the same models but exclude each individual state of residence to demonstrate the robustness of the models. Other models focus specifically on sex, race/ethnicity, and education to explore reductions in cross-state variation. These analyses are stratified in order to explore heterogeneity that may exist in the variation. Finally, two results tables are presented that exclude states which account for small portions of the sample. The first excludes states that make up less than one percent of the sample, and the second shows those with less than five percent. This is done to see if it is consequential for state of birth random effects, for they could be difficult to estimate with very small sample sizes.

In this study, Stata software version 16.1 (College Station, TX) were used for all statistical analyses. Likelihood Ratio (LR) tests were used to determine significance. All statistical tests were two-tailed, with the null hypothesis of no difference being rejected if *p* < 0.05.

## Results

Descriptive statistics of the study sample are presented in Table 1. Among the 152,373 individuals in the sample, all states, save for Alaska, are accounted for with regard to state of birth. Approximately 55,142 (36.2%) were women and 97231 (63.8%) were men, and mean age among the sample was 67.4 (SD = 1.672). Over 90% of individuals were non-Hispanic white, with those who were not non-Hispanic whites accounting for 6.6%. For education, over one-third (38.7%) had a college education or greater, while those with some college, high school, or less than high school accounted for 21.8%, 29.6%, and 7.1%, respectively.

**Table 1:** Descriptive statistics (N = 152,373).

	Total			Female	Male
	Mean	Min	Max	Mean	Mean
AD mortality	0.007	0.0	1.0	0.007	0.007
Age	67.404	65.0	70.0	67.381	67.418
Female	0.362	0.0	1.0	1.000	0.000
***Race/ethnicity***					
Non-Hispanic white	0.934	0.0	1.0	0.919	0.943
Non-Hispanic black	0.027	0.0	1.0	0.040	0.019
Non-Hispanic others	0.013	0.0	1.0	0.013	0.014
Hispanic	0.016	0.0	1.0	0.016	0.015
Missing	0.010	0.0	1.0	0.011	0.009
***Education***					
<HS	0.071	0.0	1.0	0.068	0.073
Completed high school	0.296	0.0	1.0	0.375	0.251
Some college	0.218	0.0	1.0	0.241	0.206
College graduate +	0.387	0.0	1.0	0.285	0.444
Missing	0.028	0.0	1.0	0.031	0.026
***State of residence***					
CA	0.307	0.0	1.0	0.320	0.300
FL	0.237	0.0	1.0	0.233	0.239
GA	0.022	0.0	1.0	0.023	0.022
LA	0.035	0.0	1.0	0.035	0.034
MI	0.041	0.0	1.0	0.041	0.041
NC	0.082	0.0	1.0	0.080	0.083
NJ	0.121	0.0	1.0	0.120	0.122
PA	0.155	0.0	1.0	0.149	0.159
***State of birth***					
Alabama	0.006	0.0	1.0	0.006	0.006
Alaska	0.000	0.0	1.0	0.000	0.000
Arizona	0.003	0.0	1.0	0.003	0.003
Arkansas	0.003	0.0	1.0	0.003	0.003
California	0.139	0.0	1.0	0.153	0.132
Colorado	0.005	0.0	1.0	0.005	0.005
Connecticut	0.010	0.0	1.0	0.010	0.011
Delaware	0.002	0.0	1.0	0.002	0.002
District of Columbia	0.006	0.0	1.0	0.007	0.005
Florida	0.031	0.0	1.0	0.035	0.029
Georgia	0.015	0.0	1.0	0.016	0.015
Hawaii	0.002	0.0	1.0	0.002	0.002
Idaho	0.002	0.0	1.0	0.002	0.002
Illinois	0.038	0.0	1.0	0.036	0.039
Indiana	0.014	0.0	1.0	0.013	0.015
Iowa	0.009	0.0	1.0	0.008	0.010
Kansas	0.005	0.0	1.0	0.005	0.005
Kentucky	0.006	0.0	1.0	0.005	0.006
Louisiana	0.025	0.0	1.0	0.027	0.024
Maine	0.004	0.0	1.0	0.003	0.004
Maryland	0.009	0.0	1.0	0.008	0.009
Massachusetts	0.023	0.0	1.0	0.022	0.024
Michigan	0.058	0.0	1.0	0.058	0.058
Minnesota	0.010	0.0	1.0	0.010	0.010
Mississippi	0.004	0.0	1.0	0.004	0.004
Missouri	0.011	0.0	1.0	0.010	0.011
Montana	0.002	0.0	1.0	0.001	0.002
Nebraska	0.005	0.0	1.0	0.005	0.005
Nevada	0.001	0.0	1.0	0.001	0.001
New Hampshire	0.003	0.0	1.0	0.003	0.003
New Jersey	0.096	0.0	1.0	0.098	0.094
New Mexico	0.001	0.0	1.0	0.001	0.002
New York	0.121	0.0	1.0	0.112	0.125
North Carolina	0.044	0.0	1.0	0.049	0.041
North Dakota	0.002	0.0	1.0	0.002	0.002
Ohio	0.035	0.0	1.0	0.034	0.035
Oklahoma	0.005	0.0	1.0	0.005	0.005
Oregon	0.005	0.0	1.0	0.006	0.005
Pennsylvania	0.172	0.0	1.0	0.163	0.178
Rhode Island	0.004	0.0	1.0	0.003	0.004
South Carolina	0.004	0.0	1.0	0.004	0.004
South Dakota	0.002	0.0	1.0	0.002	0.002
Tennessee	0.006	0.0	1.0	0.007	0.006
Texas	0.011	0.0	1.0	0.011	0.011
Utah	0.003	0.0	1.0	0.003	0.003
Vermont	0.002	0.0	1.0	0.002	0.002
Virginia	0.009	0.0	1.0	0.009	0.009
Washington	0.008	0.0	1.0	0.008	0.007
West Virginia	0.005	0.0	1.0	0.005	0.006
Wisconsin	0.012	0.0	1.0	0.011	0.012
Wyoming	0.001	0.0	1.0	0.001	0.001
Observations	152373			55142	97231

Note: AD = Alzheimer’s disease.

Table 2 presents the rate of concordance/discordance in state of birth and residence among our sample. For the pooled sample, about a quarter of the sample (22.79%) lived in a state that they were not born in. The discordance differs greatly by state of residence, ranging from 4.26% for California to 47.24% for Georgia.

**Table 2:** Distribution of stayers and movers, by state of residence.

	Stayers	Movers
CA	95.74	4.26
FL	84.50	15.50
GA	52.76	47.24
LA	88.24	11.76
MI	53.65	46.35
NC	87.29	12.71
NJ	69.64	30.36
PA	70.99	29.01
Total	77.21	22.79

Note: “Stayers” are those who lived in the state where they were born and “movers” are those who lived in a different state from where they were born.

The main results for all states are presented in Table 3, in the form of multi-level regression models that include both fixed and random effects in each model. Diagnostic statistics would indicate that the final model, which includes random effects of both state of birth and residence, performs best. Models 1 and 2 show that state of birth accounts for approximately 4.5% of the variance in AD mortality in the data, which is similar to results from Xu et al. (in press) for all-cause mortality using alternative data. Regarding the fixed effects, ages 65 through 68 are statistically significant across all models they are included in (models 2, 4, and 5). Meanwhile, for race/ethnicity, it shows that non-Hispanic blacks are significant across all models, whereas other racial groups are not. Models 3 and 4 then show that state of residence accounts for more than the state of birth models, at approximately 7.5% of the variation in AD mortality. However, since state of birth and state of residence are highly correlated, we estimate a final set of models that includes both random effects.

**Table 3:** Results of multilevel logistic regression models.

	(1)	(2)	(3)	(4)	(5)
	AD mortality	AD mortality	AD mortality	AD mortality	AD mortality
Age group	Full	Full	Full	Full	Full
**Fixed effects**					
Age = 65		0.414***		0.413***	0.413***
Age = 66		0.526***		0.525***	0.525***
Age = 67		0.643***		0.641***	0.641***
Age = 68		0.730**		0.729**	0.729**
Age = 69		0.847		0.845	0.845
Female		1.056		1.052	1.052
*Race/ethnicity*					
Non-Hispanic black		0.395**		0.401**	0.401**
Non-Hispanic others		0.897		0.842	0.842
Hispanic		0.815		0.785	0.785
Missing		1.008		1.000	1.000
**Random effects**					
State of birth ($\sigma_{k}^{2}$)	0.0459	0.0453			3.81e-14
State of residence ($\sigma_{j}^{2}$)			0.0765	0.0763	0.0762
N	152373	152373	152373	152373	152373
LL	-6217.0	-6169.3	-6204.5	-6156.7	-6156.7
AIC	12438.0	12362.7	12413.1	12337.4	12339.4
BIC	12457.9	12481.9	12432.9	12456.6	12468.5

Note: LL = log likelihood; AIC = Akaike information criterion; BIC = Bayesian information criterion.

* *p* < 0.05,

** *p* < 0.01,

*** *p* < 0.001.

Our final models estimate the variance of state of residence in this model to be 7.6% and show that variation from state of birth can no longer be estimated. We interpret this finding to mean that a larger proportion of the variance in the risk of AD mortality could be explained by state of residence random effects, as opposed to state of birth.

In addition to our main results, we also conducted robustness and heterogeneity analyses. Analysis stratified by social determinants of health such as sex, race/ethnicity, and education show that there is limited heterogeneity in terms of how much variation there is between state of birth and state of residence (See S2–S4 Tables). Regarding sex and race/ethnicity, analysis showed that state of residence was the predominant factor in the risk of AD mortality, similar to the main results. Education, particularly those with some college education, shows that state of birth does make up a larger share of the variance; however, this is the only instance of this occurring in all result models. For other education levels, only college education or more is responsible for some of the variation (14%) in AD mortality.

S5–S12 Tables show that our results are not driven by any particular state of residence in the analysis and are similar for sociodemographic groups. S13 and S14 Tables exclude states of birth with small numbers of respondents and show the results are unchanged. In S15 Table, we also find that the results are robust to excluding those who lived in the state where they were born.

## Discussion

Previous research has made significant contributions to the literature surrounding place-based differences in later life mortality. Despite this, much of this research has put greater emphasis on individual level factors and contemporaneous (i.e. old age) geographic contexts [4,5]. The aim of this study was to test the extent of the importance of broad-based early environments and contexts in accounting for variation in AD mortality. The analyses in this paper show that state of residence random effects explained the majority of geographic variance in the risk of AD mortality. This was the case across the main results and in supplementary robustness checks. An explanation for this could be that, while state of birth may explain health outcomes in later life as indicated in previous studies [9,15], they may not explain outcomes regarding AD mortality specifically.

These findings do partially support previous research done on how AD mortality is influenced by an individual’s level of educational attainment [31]. Further variables were to be included in the analysis, primarily those on health behaviors, but initial results suggested no need to examine those factors.

There are important limitations in this study. First, the data used for analysis only includes eight states of residence (California, Florida, Georgia, Louisiana, Michigan, New Jersey, North Carolina, and Pennsylvania). This limits the generalizability of our findings. Results from supplemental analyses suggest that no single state was responsible for the results. Future studies would benefit by employing larger sample sizes and additional states of residence. Another limitation is that our measure of early life context is state of birth rather than place of childhood. We also cannot estimate the impacts of durations of place-based exposures. Building from this, given that many people may move internally in their state of residence [13], it is difficult to ascertain the exposures that individuals faced without knowing if or when they moved. Thus, focusing on the state as the geographic unit of analysis will mask disparities within states, particularly those in which people were born and continued to reside in throughout their lives. Future studies should also examine inter-state heterogeneity to further document life course effects of exposures. A third limitation is the measurement bias of AD mortality. Many studies often use death certificates for the study of AD mortality, but often suffer from an underreporting of death due to uncertainties regarding coding practices in the cause of death [40,41]. However, when it comes to measurement of AD mortality, there is no agreed upon gold standard for measurement.

## Conclusion

This study is one of the first studies to examine the independent and joint associations of state of birth and state of residence with AD mortality. Results point to state of residence having a larger role in geographic disparities that exist in AD mortality later in life. These findings show that there should be a greater focus on contemporaneous exposures, compared to early life ones to fully ascertain what influences the likelihood death from AD. Moreover, the boarder implications of this study are that specific conditions are better explained through a place of birth lens, such as diabetes, heart disease, or cancer [5–7], while illnesses such as AD are best looked at by place of residence, in this study at least. In short, this research highlights the importance of later life factors in the examination of degenerative chronic illnesses that disproportionately impact individuals towards the end of the life span.

## Supporting information

S1 Table Sample inclusion tests. (DOCX)

S2 Table Heterogeneity: Sex. (DOCX)

S3 Table Heterogeneity: Race/Ethnicity. (DOCX)

S4 Table Heterogeneity: Education. (DOCX)

S5 Table Robustness: Excluding CA. (DOCX)

S6 Table Robustness: Excluding FL. (DOCX)

S7 Table Robustness: Excluding GA. (DOCX)

S8 Table Robustness: Excluding LA. (DOCX)

S9 Table Robustness: Excluding MI. (DOCX)

S10 Table Robustness: Excluding NC. (DOCX)

S11 Table Robustness: Excluding NJ. (DOCX)

S12 Table Robustness: Excluding PA. (DOCX)

S13 Table Robustness: Excluding states of birth with less than 1% of the total sample. (DOCX)

S14 Table Robustness: Excluding states of birth with less than 5% of the total sample. (DOCX)

S15 Table Robustness: Excluding those who lived in the state where they were born. (DOCX)
