# Supplementary material for: Geographic variation in Alzheimer’s disease mortality
Source: PLoS One. 2021 Jul 1;16(7):e0254174. doi: 10.1371/journal.pone.0254174 (PMC8248693; doi:10.1371/journal.pone.0254174)
Supplement: S1 Table — (DOCX) [file pone.0254174.s001.docx]

# S1 Table. Sample Inclusion Tests

|  | (1) |
| --- | --- |
|  | Being in the analytic sample |
| Age | 2.557^***^ |
| Female | 0.994 |
| *Race/ethnicity* |  |
| Non-Hispanic black | 0.955 |
| Non-Hispanic others | 0.877^*^ |
| Hispanic | 1.014 |
| Missing | 0.965 |
| *Education* |  |
| Completed high school | 1.047 |
| Some college | 1.042 |
| College graduate + | 1.036 |
| Missing | 1.002 |
| N | 400481 |
| LL | -85482.3 |
| AIC | 170986.7 |
| BIC | 171106.6 |

^*^ *p* < 0.05, ^**^ *p* < 0.01, ^***^ *p* < 0.001
