# Supplementary material for: Geographic variation in Alzheimer’s disease mortality
Source: PLoS One. 2021 Jul 1;16(7):e0254174. doi: 10.1371/journal.pone.0254174 (PMC8248693; doi:10.1371/journal.pone.0254174)
Supplement: S2 Table — (DOCX) [file pone.0254174.s002.docx]

# S2 Table. Heterogeneity: Sex

|  | (1) | (2) |
| --- | --- | --- |
|  | AD mortality | AD mortality |
| Heterogenous group | Male | Female |
| **Fixed effects** |  |  |
| Age = 65 | 0.408^***^ | 0.423^***^ |
| Age = 66 | 0.497^***^ | 0.574^**^ |
| Age = 67 | 0.644^***^ | 0.636^**^ |
| Age = 68 | 0.747^*^ | 0.698^*^ |
| Age = 69 | 0.796 | 0.934 |
| Female |  |  |
| *Race/ethnicity* |  |  |
| Non-Hispanic black | 0.325^*^ | 0.469^*^ |
| Non-Hispanic others | 0.667 | 1.165 |
| Hispanic | 0.815 | 0.743 |
| Missing | 0.928 | 1.102 |
| **Random effects** |  |  |
| State of birth ($\sigma_{k}^{2})$ | 4.21e-11 | 7.65e-10 |
| State of residence ($\sigma_{j}^{2})$ | 0.0645 | 0.0772 |
| N | 97231 | 55142 |
| LL | -3880.3 | -2279.8 |
| AIC | 7784.5 | 4583.5 |
| BIC | 7898.3 | 4690.5 |

^*^ *p* < 0.05, ^**^ *p* < 0.01, ^***^ *p* < 0.001
