# Supplementary material for: Geographic variation in Alzheimer’s disease mortality
Source: PLoS One. 2021 Jul 1;16(7):e0254174. doi: 10.1371/journal.pone.0254174 (PMC8248693; doi:10.1371/journal.pone.0254174)
Supplement: S3 Table — (DOCX) [file pone.0254174.s003.docx]

# S3 Table. Heterogeneity: Race/Ethnicity

|  | (1) | (2) |
| --- | --- | --- |
|  | AD mortality | AD mortality |
| Heterogenous group | Non-Hispanic white = Yes | Non-Hispanic white = No |
| **Fixed effects** |  |  |
| Age = 65 | 0.443^***^ | 0.0452^**^ |
| Age = 66 | 0.537^***^ | 0.344^*^ |
| Age = 67 | 0.652^***^ | 0.482 |
| Age = 68 | 0.751^**^ | 0.434^*^ |
| Age = 69 | 0.882 | 0.358^*^ |
| Female | 1.039 | 1.224 |
| **Random effects** |  |  |
| State of birth ($\sigma_{k}^{2})$ | 1.06e-12 | 3.04e-20 |
| State of residence ($\sigma_{j}^{2})$ | 0.0759 | 0.196 |
| N | 142358 | 10015 |
| LL | -5852.3 | -303.0 |
| AIC | 11722.7 | 623.9 |
| BIC | 11811.5 | 688.8 |

^*^ *p* < 0.05, ^**^ *p* < 0.01, ^***^ *p* < 0.001
