# Supplementary material for: Geographic variation in Alzheimer’s disease mortality
Source: PLoS One. 2021 Jul 1;16(7):e0254174. doi: 10.1371/journal.pone.0254174 (PMC8248693; doi:10.1371/journal.pone.0254174)
Supplement: S4 Table — (DOCX) [file pone.0254174.s004.docx]

# S4 Table. Heterogeneity: Education

|  | (1) | (2) | (3) | (4) | (5) |
| --- | --- | --- | --- | --- | --- |
|  | AD mortality | AD mortality | AD mortality | AD mortality | AD mortality |
| Heterogenous group | <HS | Completed HS | Some college | College graduate + | Missing |
| **Fixed effects** |  |  |  |  |  |
| Age = 65 | 0.193^**^ | 0.375^***^ | 0.586^*^ | 0.378^***^ | 0.590 |
| Age = 66 | 0.531 | 0.576^**^ | 0.612^*^ | 0.452^***^ | 0.447 |
| Age = 67 | 0.134^**^ | 0.677^*^ | 0.596^*^ | 0.751 | 0.844 |
| Age = 68 | 0.786 | 0.621^*^ | 0.612^*^ | 0.876 | 0.617 |
| Age = 69 | 0.319^**^ | 0.966 | 0.863 | 0.914 | 0.724 |
| Female | 0.982 | 1.197 | 0.849 | 1.167 | 0.846 |
| *Race/ethnicity* |  |  |  |  |  |
| Non-Hispanic black | 0.292 | 0.141 | 0.606 | 0.436 | 0.574 |
| Non-Hispanic others | 1.171 | 0.865 | 1.365 | 0.728 | 0.00000157 |
| Hispanic | 0.419 | 1.710 | 0.769 | 0.394 | 0.00000124 |
| Missing | 4.49e-08 | 0.820 | 1.447 | 1.106 | 0.844 |
| **Random effects** |  |  |  |  |  |
| State of birth ($\sigma_{k}^{2})$ | 2.95e-12 | 2.77e-19 | 0.00410 | 0.0119 | 0.000000383 |
| State of residence ($\sigma_{j}^{2})$ | 0.0699 | 0.0713 | 3.21e-17 | 0.0740 | 0.136 |
| N | 10812 | 45102 | 33270 | 58912 | 4277 |
| LL | -405.8 | -1682.9 | -1435.0 | -2392.3 | -218.5 |
| AIC | 837.6 | 3391.8 | 2896.0 | 4810.6 | 463.1 |
| BIC | 932.3 | 3505.1 | 3005.3 | 4927.4 | 545.8 |

^*^ *p* < 0.05, ^**^ *p* < 0.01, ^***^ *p* < 0.001
