# Supplementary material for: Geographic variation in Alzheimer’s disease mortality
Source: PLoS One. 2021 Jul 1;16(7):e0254174. doi: 10.1371/journal.pone.0254174 (PMC8248693; doi:10.1371/journal.pone.0254174)
Supplement: S5 Table — (DOCX) [file pone.0254174.s005.docx]

# S5 Table. Robustness: Excluding CA

|  | (1) | (2) | (3) | (4) | (5) |
| --- | --- | --- | --- | --- | --- |
|  | AD mortality | AD mortality | AD mortality | AD mortality | AD mortality |
| **Fixed effects** |  |  |  |  |  |
| Age = 65 |  | 0.445^***^ |  | 0.445^***^ | 0.445^***^ |
| Age = 66 |  | 0.514^***^ |  | 0.513^***^ | 0.513^***^ |
| Age = 67 |  | 0.637^***^ |  | 0.637^***^ | 0.637^***^ |
| Age = 68 |  | 0.691^**^ |  | 0.690^**^ | 0.690^**^ |
| Age = 69 |  | 0.765^*^ |  | 0.764^*^ | 0.764^*^ |
| Female |  | 1.042 |  | 1.042 | 1.042 |
| *Race/ethnicity* |  |  |  |  |  |
| Non-Hispanic black |  | 0.362^**^ |  | 0.366^**^ | 0.366^**^ |
| Non-Hispanic others |  | 0.932 |  | 0.967 | 0.967 |
| Hispanic |  | 0.634 |  | 0.618 | 0.618 |
| Missing |  | 0.938 |  | 0.941 | 0.941 |
| **Random effects** |  |  |  |  |  |
| State of birth ($\sigma_{k}^{2})$ | 0.0409 | 0.0415 |  |  | 6.57e-14 |
| State of residence ($\sigma_{j}^{2})$ |  |  | 0.0706 | 0.0709 | 0.0708 |
| N | 105536 | 105536 | 105536 | 105536 | 105536 |
| LL | -3844.8 | -3818.3 | -3841.7 | -3815.3 | -3815.3 |
| AIC | 7693.6 | 7660.6 | 7687.5 | 7654.6 | 7656.6 |
| BIC | 7712.7 | 7775.4 | 7706.6 | 7769.4 | 7781.0 |

^*^ *p* < 0.05, ^**^ *p* < 0.01, ^***^ *p* < 0.001
