# Supplementary material for: Geographic variation in Alzheimer’s disease mortality
Source: PLoS One. 2021 Jul 1;16(7):e0254174. doi: 10.1371/journal.pone.0254174 (PMC8248693; doi:10.1371/journal.pone.0254174)
Supplement: S6 Table — (DOCX) [file pone.0254174.s006.docx]

# S6 Table. Robustness: Excluding FL

|  | (1) | (2) | (3) | (4) | (5) |
| --- | --- | --- | --- | --- | --- |
|  | AD mortality | AD mortality | AD mortality | AD mortality | AD mortality |
| **Fixed effects** |  |  |  |  |  |
| Age = 65 |  | 0.416^***^ |  | 0.416^***^ | 0.416^***^ |
| Age = 66 |  | 0.539^***^ |  | 0.538^***^ | 0.538^***^ |
| Age = 67 |  | 0.670^***^ |  | 0.668^***^ | 0.668^***^ |
| Age = 68 |  | 0.747^*^ |  | 0.747^*^ | 0.747^*^ |
| Age = 69 |  | 0.895 |  | 0.893 | 0.893 |
| Female |  | 1.024 |  | 1.022 | 1.022 |
| *Race/ethnicity* |  |  |  |  |  |
| Non-Hispanic black |  | 0.354^**^ |  | 0.372^**^ | 0.372^**^ |
| Non-Hispanic others |  | 0.871 |  | 0.830 | 0.829 |
| Hispanic |  | 0.855 |  | 0.823 | 0.823 |
| Missing |  | 0.895 |  | 0.892 | 0.893 |
| **Random effects** |  |  |  |  |  |
| State of birth ($\sigma_{k}^{2})$ | 0.0538 | 0.0545 |  |  | 0.00278 |
| State of residence ($\sigma_{j}^{2})$ |  |  | 0.0910 | 0.0898 | 0.0884 |
| N | 116321 | 116321 | 116321 | 116321 | 116321 |
| LL | -4873.6 | -4833.8 | -4861.9 | -4822.6 | -4822.6 |
| AIC | 9751.3 | 9691.6 | 9727.8 | 9669.2 | 9671.2 |
| BIC | 9770.6 | 9807.6 | 9747.1 | 9785.2 | 9796.8 |

^*^ *p* < 0.05, ^**^ *p* < 0.01, ^***^ *p* < 0.001
