# Supplementary material for: Geographic variation in Alzheimer’s disease mortality
Source: PLoS One. 2021 Jul 1;16(7):e0254174. doi: 10.1371/journal.pone.0254174 (PMC8248693; doi:10.1371/journal.pone.0254174)
Supplement: S7 Table — (DOCX) [file pone.0254174.s007.docx]

# S7 Table. Robustness: Excluding GA

|  | (1) | (2) | (3) | (4) | (5) |
| --- | --- | --- | --- | --- | --- |
|  | AD mortality | AD mortality | AD mortality | AD mortality | AD mortality |
| **Fixed effects** |  |  |  |  |  |
| Age = 65 |  | 0.421^***^ |  | 0.419^***^ | 0.419^***^ |
| Age = 66 |  | 0.533^***^ |  | 0.533^***^ | 0.533^***^ |
| Age = 67 |  | 0.642^***^ |  | 0.639^***^ | 0.639^***^ |
| Age = 68 |  | 0.737^**^ |  | 0.736^**^ | 0.736^**^ |
| Age = 69 |  | 0.852 |  | 0.850 | 0.850 |
| Female |  | 1.068 |  | 1.064 | 1.064 |
| *Race/ethnicity* |  |  |  |  |  |
| Non-Hispanic black |  | 0.411^**^ |  | 0.415^**^ | 0.415^**^ |
| Non-Hispanic others |  | 0.898 |  | 0.845 | 0.845 |
| Hispanic |  | 0.819 |  | 0.788 | 0.788 |
| Missing |  | 1.030 |  | 1.021 | 1.021 |
| **Random effects** |  |  |  |  |  |
| State of birth ($\sigma_{k}^{2})$ | 0.0522 | 0.0516 |  |  | 1.21e-11 |
| State of residence ($\sigma_{j}^{2})$ |  |  | 0.0737 | 0.0751 | 0.0750 |
| N | 148947 | 148947 | 148947 | 148947 | 148947 |
| LL | -6117.9 | -6072.5 | -6107.1 | -6061.3 | -6061.3 |
| AIC | 12239.8 | 12169.1 | 12218.1 | 12146.7 | 12148.7 |
| BIC | 12259.6 | 12288.0 | 12237.9 | 12265.6 | 12277.5 |

^*^ *p* < 0.05, ^**^ *p* < 0.01, ^***^ *p* < 0.001
