# Supplementary material for: Geographic variation in Alzheimer’s disease mortality
Source: PLoS One. 2021 Jul 1;16(7):e0254174. doi: 10.1371/journal.pone.0254174 (PMC8248693; doi:10.1371/journal.pone.0254174)
Supplement: S8 Table — (DOCX) [file pone.0254174.s008.docx]

# S8 Table. Robustness: Excluding LA

|  | (1) | (2) | (3) | (4) | (5) |
| --- | --- | --- | --- | --- | --- |
|  | AD mortality | AD mortality | AD mortality | AD mortality | AD mortality |
| **Fixed effects** |  |  |  |  |  |
| Age = 65 |  | 0.399^***^ |  | 0.398^***^ | 0.398^***^ |
| Age = 66 |  | 0.522^***^ |  | 0.522^***^ | 0.522^***^ |
| Age = 67 |  | 0.649^***^ |  | 0.647^***^ | 0.647^***^ |
| Age = 68 |  | 0.721^**^ |  | 0.720^**^ | 0.720^**^ |
| Age = 69 |  | 0.864 |  | 0.863 | 0.863 |
| Female |  | 1.056 |  | 1.052 | 1.052 |
| *Race/ethnicity* |  |  |  |  |  |
| Non-Hispanic black |  | 0.401^**^ |  | 0.403^**^ | 0.403^**^ |
| Non-Hispanic others |  | 0.931 |  | 0.867 | 0.867 |
| Hispanic |  | 0.847 |  | 0.812 | 0.812 |
| Missing |  | 1.084 |  | 1.074 | 1.074 |
| **Random effects** |  |  |  |  |  |
| State of birth ($\sigma_{k}^{2})$ | 0.0367 | 0.0347 |  |  | 3.49e-17 |
| State of residence ($\sigma_{j}^{2})$ |  |  | 0.0427 | 0.0425 | 0.0425 |
| N | 147116 | 147116 | 147116 | 147116 | 147116 |
| LL | -5880.0 | -5832.7 | -5868.7 | -5821.2 | -5821.2 |
| AIC | 11763.9 | 11689.3 | 11741.5 | 11666.4 | 11668.4 |
| BIC | 11783.7 | 11808.1 | 11761.3 | 11785.1 | 11797.0 |

^*^ *p* < 0.05, ^**^ *p* < 0.01, ^***^ *p* < 0.001
