# Supplementary material for: Geographic variation in Alzheimer’s disease mortality
Source: PLoS One. 2021 Jul 1;16(7):e0254174. doi: 10.1371/journal.pone.0254174 (PMC8248693; doi:10.1371/journal.pone.0254174)
Supplement: S9 Table — (DOCX) [file pone.0254174.s009.docx]

# S9 Table. Robustness: Excluding MI

|  | (1) | (2) | (3) | (4) | (5) |
| --- | --- | --- | --- | --- | --- |
|  | AD mortality | AD mortality | AD mortality | AD mortality | AD mortality |
| **Fixed effects** |  |  |  |  |  |
| Age = 65 |  | 0.430^***^ |  | 0.428^***^ | 0.428^***^ |
| Age = 66 |  | 0.540^***^ |  | 0.539^***^ | 0.539^***^ |
| Age = 67 |  | 0.659^***^ |  | 0.656^***^ | 0.656^***^ |
| Age = 68 |  | 0.751^**^ |  | 0.750^**^ | 0.750^**^ |
| Age = 69 |  | 0.847 |  | 0.845 | 0.845 |
| Female |  | 1.045 |  | 1.041 | 1.041 |
| *Race/ethnicity* |  |  |  |  |  |
| Non-Hispanic black |  | 0.442^**^ |  | 0.445^**^ | 0.445^**^ |
| Non-Hispanic others |  | 0.909 |  | 0.853 | 0.853 |
| Hispanic |  | 0.825 |  | 0.793 | 0.793 |
| Missing |  | 1.055 |  | 1.044 | 1.044 |
| **Random effects** |  |  |  |  |  |
| State of birth ($\sigma_{k}^{2})$ | 0.0474 | 0.0466 |  |  | 3.38e-14 |
| State of residence ($\sigma_{j}^{2})$ |  |  | 0.0879 | 0.0880 | 0.0879 |
| N | 146159 | 146159 | 146159 | 146159 | 146159 |
| LL | -5985.2 | -5944.1 | -5972.2 | -5930.8 | -5930.8 |
| AIC | 11974.5 | 11912.2 | 11948.5 | 11885.6 | 11887.6 |
| BIC | 11994.3 | 12030.9 | 11968.2 | 12004.3 | 12016.2 |

^*^ *p* < 0.05, ^**^ *p* < 0.01, ^***^ *p* < 0.001
