# Supplementary material for: Geographic variation in Alzheimer’s disease mortality
Source: PLoS One. 2021 Jul 1;16(7):e0254174. doi: 10.1371/journal.pone.0254174 (PMC8248693; doi:10.1371/journal.pone.0254174)
Supplement: S10 Table — (DOCX) [file pone.0254174.s010.docx]

# S10 Table. Robustness: Excluding NC

|  | (1) | (2) | (3) | (4) | (5) |
| --- | --- | --- | --- | --- | --- |
|  | AD mortality | AD mortality | AD mortality | AD mortality | AD mortality |
| **Fixed effects** |  |  |  |  |  |
| Age = 65 |  | 0.407^***^ |  | 0.405^***^ | 0.405^***^ |
| Age = 66 |  | 0.498^***^ |  | 0.497^***^ | 0.497^***^ |
| Age = 67 |  | 0.646^***^ |  | 0.643^***^ | 0.643^***^ |
| Age = 68 |  | 0.771^*^ |  | 0.770^*^ | 0.770^*^ |
| Age = 69 |  | 0.834 |  | 0.833 | 0.833 |
| Female |  | 1.087 |  | 1.082 | 1.082 |
| *Race/ethnicity* |  |  |  |  |  |
| Non-Hispanic black |  | 0.360^**^ |  | 0.367^**^ | 0.367^**^ |
| Non-Hispanic others |  | 0.857 |  | 0.796 | 0.796 |
| Hispanic |  | 0.828 |  | 0.795 | 0.795 |
| Missing |  | 1.093 |  | 1.084 | 1.084 |
| **Random effects** |  |  |  |  |  |
| State of birth ($\sigma_{k}^{2})$ | 0.0496 | 0.0487 |  |  | 1.40e-11 |
| State of residence ($\sigma_{j}^{2})$ |  |  | 0.0900 | 0.0895 | 0.0894 |
| N | 139931 | 139931 | 139931 | 139931 | 139931 |
| LL | -5698.5 | -5650.5 | -5685.0 | -5636.7 | -5636.7 |
| AIC | 11401.1 | 11325.0 | 11373.9 | 11297.5 | 11299.5 |
| BIC | 11420.8 | 11443.2 | 11393.6 | 11415.7 | 11427.5 |

^*^ *p* < 0.05, ^**^ *p* < 0.01, ^***^ *p* < 0.001
