# Supplementary material for: Geographic variation in Alzheimer’s disease mortality
Source: PLoS One. 2021 Jul 1;16(7):e0254174. doi: 10.1371/journal.pone.0254174 (PMC8248693; doi:10.1371/journal.pone.0254174)
Supplement: S11 Table — (DOCX) [file pone.0254174.s011.docx]

# S11 Table. Robustness: Excluding NJ

|  | (1) | (2) | (3) | (4) | (5) |
| --- | --- | --- | --- | --- | --- |
|  | AD mortality | AD mortality | AD mortality | AD mortality | AD mortality |
| **Fixed effects** |  |  |  |  |  |
| Age = 65 |  | 0.410^***^ |  | 0.408^***^ | 0.408^***^ |
| Age = 66 |  | 0.522^***^ |  | 0.521^***^ | 0.521^***^ |
| Age = 67 |  | 0.636^***^ |  | 0.633^***^ | 0.633^***^ |
| Age = 68 |  | 0.732^**^ |  | 0.730^**^ | 0.730^**^ |
| Age = 69 |  | 0.871 |  | 0.870 | 0.870 |
| Female |  | 1.067 |  | 1.063 | 1.063 |
| *Race/ethnicity* |  |  |  |  |  |
| Non-Hispanic black |  | 0.443^**^ |  | 0.443^**^ | 0.443^**^ |
| Non-Hispanic others |  | 0.874 |  | 0.819 | 0.819 |
| Hispanic |  | 0.841 |  | 0.820 | 0.820 |
| Missing |  | 1.094 |  | 1.084 | 1.084 |
| **Random effects** |  |  |  |  |  |
| State of birth ($\sigma_{k}^{2})$ | 0.0390 | 0.0382 |  |  | 1.83e-15 |
| State of residence ($\sigma_{j}^{2})$ |  |  | 0.0645 | 0.0642 | 0.0641 |
| N | 133869 | 133869 | 133869 | 133869 | 133869 |
| LL | -5674.0 | -5629.1 | -5665.7 | -5620.2 | -5620.2 |
| AIC | 11352.0 | 11282.1 | 11335.3 | 11264.4 | 11266.4 |
| BIC | 11371.6 | 11399.8 | 11354.9 | 11382.1 | 11393.9 |

^*^ *p* < 0.05, ^**^ *p* < 0.01, ^***^ *p* < 0.001
