# Supplementary material for: Geographic variation in Alzheimer’s disease mortality
Source: PLoS One. 2021 Jul 1;16(7):e0254174. doi: 10.1371/journal.pone.0254174 (PMC8248693; doi:10.1371/journal.pone.0254174)
Supplement: S12 Table — (DOCX) [file pone.0254174.s012.docx]

# S12 Table. Robustness: Excluding PA

|  | (1) | (2) | (3) | (4) | (5) |
| --- | --- | --- | --- | --- | --- |
|  | AD mortality | AD mortality | AD mortality | AD mortality | AD mortality |
| **Fixed effects** |  |  |  |  |  |
| Age = 65 |  | 0.397^***^ |  | 0.396^***^ | 0.396^***^ |
| Age = 66 |  | 0.534^***^ |  | 0.534^***^ | 0.534^***^ |
| Age = 67 |  | 0.609^***^ |  | 0.607^***^ | 0.607^***^ |
| Age = 68 |  | 0.681^***^ |  | 0.680^***^ | 0.680^***^ |
| Age = 69 |  | 0.825 |  | 0.824 | 0.824 |
| Female |  | 1.050 |  | 1.046 | 1.046 |
| *Race/ethnicity* |  |  |  |  |  |
| Non-Hispanic black |  | 0.382^**^ |  | 0.383^**^ | 0.383^**^ |
| Non-Hispanic others |  | 0.912 |  | 0.860 | 0.860 |
| Hispanic |  | 0.764 |  | 0.739 | 0.739 |
| Missing |  | 0.825 |  | 0.814 | 0.814 |
| **Random effects** |  |  |  |  |  |
| State of birth ($\sigma_{k}^{2})$ | 0.0454 | 0.0440 |  |  | 1.80e-09 |
| State of residence ($\sigma_{j}^{2})$ |  |  | 0.0812 | 0.0799 | 0.0798 |
| N | 128732 | 128732 | 128732 | 128732 | 128732 |
| LL | -5438.3 | -5394.6 | -5428.3 | -5384.3 | -5384.3 |
| AIC | 10880.7 | 10813.2 | 10860.6 | 10792.5 | 10794.5 |
| BIC | 10900.2 | 10930.4 | 10880.1 | 10909.7 | 10921.5 |

^*^ *p* < 0.05, ^**^ *p* < 0.01, ^***^ *p* < 0.001
