# Supplementary material for: Geographic variation in Alzheimer’s disease mortality
Source: PLoS One. 2021 Jul 1;16(7):e0254174. doi: 10.1371/journal.pone.0254174 (PMC8248693; doi:10.1371/journal.pone.0254174)
Supplement: S13 Table — (DOCX) [file pone.0254174.s013.docx]

# S13 Table. Robustness: Excluding States of Birth with Less Than 1% of the Total Sample

|  | (1) | (2) | (3) | (4) | (5) |
| --- | --- | --- | --- | --- | --- |
|  | AD mortality | AD mortality | AD mortality | AD mortality | AD mortality |
| **Fixed effects** |  |  |  |  |  |
| Age = 65 |  | 0.392^***^ |  | 0.390^***^ | 0.390^***^ |
| Age = 66 |  | 0.519^***^ |  | 0.519^***^ | 0.519^***^ |
| Age = 67 |  | 0.650^***^ |  | 0.648^***^ | 0.648^***^ |
| Age = 68 |  | 0.740^**^ |  | 0.739^**^ | 0.739^**^ |
| Age = 69 |  | 0.863 |  | 0.861 | 0.861 |
| Female |  | 1.108 |  | 1.105 | 1.105 |
| *Race/ethnicity* |  |  |  |  |  |
| Non-Hispanic black |  | 0.313^**^ |  | 0.311^**^ | 0.311^**^ |
| Non-Hispanic others |  | 0.606 |  | 0.592 | 0.592 |
| Hispanic |  | 0.841 |  | 0.830 | 0.830 |
| Missing |  | 1.112 |  | 1.104 | 1.104 |
| **Random effects** |  |  |  |  |  |
| State of birth ($\sigma_{k}^{2})$ | 0.0455 | 0.0454 |  |  | 6.29e-14 |
| State of residence ($\sigma_{j}^{2})$ |  |  | 0.0661 | 0.0680 | 0.0679 |
| N | 131768 | 131768 | 131768 | 131768 | 131768 |
| LL | -5180.0 | -5133.7 | -5172.9 | -5126.2 | -5126.2 |
| AIC | 10363.9 | 10291.3 | 10349.9 | 10276.4 | 10278.4 |
| BIC | 10383.5 | 10408.8 | 10369.5 | 10393.8 | 10405.6 |

^*^ *p* < 0.05, ^**^ *p* < 0.01, ^***^ *p* < 0.001
