# Supplementary material for: Geographic variation in Alzheimer’s disease mortality
Source: PLoS One. 2021 Jul 1;16(7):e0254174. doi: 10.1371/journal.pone.0254174 (PMC8248693; doi:10.1371/journal.pone.0254174)
Supplement: S14 Table — (DOCX) [file pone.0254174.s014.docx]

# S14 Table. Robustness: Excluding States of Birth with Less Than 5% of the Total Sample

|  | (1) | (2) | (3) | (4) | (5) |
| --- | --- | --- | --- | --- | --- |
|  | AD mortality | AD mortality | AD mortality | AD mortality | AD mortality |
| **Fixed effects** |  |  |  |  |  |
| Age = 65 |  | 0.316^***^ |  | 0.317^***^ | 0.317^***^ |
| Age = 66 |  | 0.495^***^ |  | 0.496^***^ | 0.496^***^ |
| Age = 67 |  | 0.668^**^ |  | 0.668^**^ | 0.668^**^ |
| Age = 68 |  | 0.733^*^ |  | 0.734^*^ | 0.734^*^ |
| Age = 69 |  | 0.838 |  | 0.837 | 0.837 |
| Female |  | 1.105 |  | 1.104 | 1.104 |
| *Race/ethnicity* |  |  |  |  |  |
| Non-Hispanic black |  | 0.349^*^ |  | 0.356^*^ | 0.356^*^ |
| Non-Hispanic others |  | 0.767 |  | 0.753 | 0.753 |
| Hispanic |  | 1.153 |  | 1.141 | 1.141 |
| Missing |  | 1.028 |  | 1.028 | 1.028 |
| **Random effects** |  |  |  |  |  |
| State of birth ($\sigma_{k}^{2})$ | 0.0309 | 0.0310 |  |  | 1.46e-14 |
| State of residence ($\sigma_{j}^{2})$ |  |  | 0.0434 | 0.0422 | 0.0422 |
| N | 89248 | 89248 | 89248 | 89248 | 89248 |
| LL | -3442.6 | -3406.2 | -3437.3 | -3401.2 | -3401.2 |
| AIC | 6889.3 | 6836.4 | 6878.6 | 6826.4 | 6828.4 |
| BIC | 6908.1 | 6949.2 | 6897.4 | 6939.2 | 6950.6 |

^*^ *p* < 0.05, ^**^ *p* < 0.01, ^***^ *p* < 0.001
