# Supplementary material for: Geographic variation in Alzheimer’s disease mortality
Source: PLoS One. 2021 Jul 1;16(7):e0254174. doi: 10.1371/journal.pone.0254174 (PMC8248693; doi:10.1371/journal.pone.0254174)
Supplement: S15 Table — (DOCX) [file pone.0254174.s015.docx]

# S15 Table. Robustness: Excluding Those Who Lived in the State Where They Were Born

|  | (1) | (2) | (3) | (4) | (5) |
| --- | --- | --- | --- | --- | --- |
|  | AD mortality | AD mortality | AD mortality | AD mortality | AD mortality |
| **Fixed effects** |  |  |  |  |  |
| Age = 65 |  | 0.415^***^ |  | 0.414^***^ | 0.414^***^ |
| Age = 66 |  | 0.521^***^ |  | 0.522^***^ | 0.522^***^ |
| Age = 67 |  | 0.649^**^ |  | 0.647^**^ | 0.647^**^ |
| Age = 68 |  | 0.775 |  | 0.775 | 0.775 |
| Age = 69 |  | 0.845 |  | 0.846 | 0.846 |
| Female |  | 1.057 |  | 1.052 | 1.052 |
| *Race/ethnicity* |  |  |  |  |  |
| Non-Hispanic black |  | 0.619 |  | 0.612 | 0.612 |
| Non-Hispanic others |  | 1.201 |  | 1.091 | 1.091 |
| Hispanic |  | 0.553 |  | 0.534 | 0.538 |
| Missing |  | 0.993 |  | 0.981 | 0.983 |
| **Random effects** |  |  |  |  |  |
| State of birth ($\sigma_{k}^{2})$ | 0.00672 | 0.00640 |  |  | 0.00289 |
| State of residence ($\sigma_{j}^{2})$ |  |  | 0.0293 | 0.0285 | 0.0280 |
| N | 84096 | 84096 | 84096 | 84096 | 84096 |
| LL | -3543.5 | -3517.6 | -3539.0 | -3513.0 | -3512.9 |
| AIC | 7091.0 | 7059.2 | 7082.0 | 7049.9 | 7051.8 |
| BIC | 7109.6 | 7171.2 | 7100.7 | 7162.0 | 7173.2 |

^*^ *p* < 0.05, ^**^ *p* < 0.01, ^***^ *p* < 0.001
